# Bi-LSTM-Augmented Deep Neural Network for Multi-Gbps VCSEL-Based Visible Light Communication Link

**DOI:** 10.3390/s22114145

**Published:** 2022-05-30

**Authors:** Seoyeon Oh, Minseok Yu, Seonghyeon Cho, Song Noh, Hyunchae Chun

**Affiliations:** Department of Information and Telecommunication Engineering, Incheon National University, Incheon 22012, Korea; seoyeon.oh@inu.ac.kr (S.O.); alseoddl852@inu.ac.kr (M.Y.); seonghyeon.cho@inu.ac.kr (S.C.)

**Keywords:** visible light communication (VLC), machine learning (ML), optical wireless communication (OWC), long short-term memory (LSTM)

## Abstract

With the remarkable advances in vertical-cavity surface-emitting lasers (VCSELs) in recent decades, VCSELs have been considered promising light sources in the field of optical wireless communications. However, off-the-shelf VCSELs still have a limited modulation bandwidth to meet the multi-Gb/s data rate requirements imposed on the next-generation wireless communication system. Recently, employing machine learning (ML) techniques as a method to tackle such issues has been intriguing for researchers in wireless communication. In this work, through a systematic analysis, it is shown that the ML technique is also very effective in VCSEL-based visible light communication. Using a commercial VCSEL and bidirectional long short-term memory (Bi-LSTM)-based ML scheme, a high-speed visible light communication (VLC) link with a data rate of 13.5 Gbps is demonstrated, which is the fastest single channel result from a cost-effective, off-the-shelf VCSEL device, to the best of the authors’ knowledge.

## 1. Introduction

With the advent of big-data technology and extremely fast computing speeds, machine learning for data-intensive science has developed. Machine learning (ML) is a field of artificial intelligent (AI) that automates the generation of models for data analysis, allowing software to learn and find patterns based on data. This helps minimize human intervention and accelerate decision making. In recent years, machine learning has served as a useful technology in various fields such as gender recognition from facial images for security systems or mobile devices [1], the analysis of financial fraud detection [2] and the prediction of biological data [3]. Additionally, it is shown that machine learning is useful in many areas of the agriculture industry, including crop management, livestock management, water management and soil management [4]. Machine learning can contribute to both the physical layer and the network layer in the field of optical communication as well [5,6,7]. It can also improve the performances in diverse fields such as physics [8], chemistry [9], society [10], economy [11] and communication [7]. More introductory information about the basic ideas of machine learning methods can be found in [12]. With the emergence of the next generation communication networks, there has been a significant interest in machine learning as a technology that can realize next-generation communication systems. Adversarial machine learning (AML) in radio frequency (RF) wireless communications has been studied for defending against cyberattacks, eventually developing a secure system for the foundation of next-generation wireless communications [13,14].

To meet the requirements imposed on the next-generation wireless communication, data rates that are an order of magnitude higher and have substantially lower latency, compared to the existing network, should be provided. Optical wireless communication (OWC) and visible light communication (VLC) have been attractive solutions mainly due to their license-free and high spectral efficiency [15]. There are two types of light sources used in most OWC and VLC systems: light-emitting diodes (LED) and laser diodes (LD). LEDs have a broad spectral width, and they have been widely deployed as lighting sources due to their cost-effectiveness and high efficacy. LDs have a narrow spectral and can be utilized to achieve higher data rates and longer transmission distances in VLC systems thanks to their coherent characteristics. However, LDs are relatively expensive sources and often require sophisticated temperature control. The modulation bandwidth of LEDs, in general, ranges from a few MHz to tens of MHz, and that of LDs is orders of magnitude higher. Additionally, it is useful to apply wideband auxiliary components to avoid the additional bandwidth limitation on the light sources [16]. Using a single LED, a VLC communication link with a data rate of 8 Gbps was reported [17], where a resonant cavity LED with direct current-biased orthogonal frequency division multiplexing (DCO-OFDM) was used. Employing a single GaN-based blue laser diode, the authors in [18] achieved a data rate of 15 Gbps at a distance of 15 cm.

With the advancement of vertical-cavity surface-emitting laser (VCSEL) diodes in recent decades, researchers have been exploring the potential of VCSEL as a light source for OWC and VLC systems. VCSELs are cost-effective compared to other LDs and have a faster modulation bandwidth than LEDs. They require a low driving current and provide an excellent beam quality with a circular emission pattern, suitable for OWC and VLC [19]. Further, VCSELs’ short cavity results in a single longitudinal mode only. Therefore, VCSELs do not suffer from kink phenomena in the optical L-I curve, whereas other semiconductor lasers suffer from power instability and control problems [20]. A single channel data rate of 12.5 Gbps using a VCSEL with 5.2 GHz of bandwidth was demonstrated with 16-level quadrature amplitude modulation (QAM) OFDM modulation [21]. A bi-directional wavelength remodulated single VCSEL link with a data rate of 10.6 Gbps for downstream activity and 2 Mbps for upstream activity was demonstrated in [22]. However, typical cost-effective commercial VCSELs have a sub–GHz modulation bandwidth. Considering the demands imposed upon the efficient next-generation wireless network capable of data rates beyond 10 Gbps, a low-cost VCSEL wireless link with advanced communication schemes should be investigated.

More recently, there has been considerable interest in improving communication quality by utilizing machine learning for VLC. In [23], a machine-learning-based low-cost visible light positioning system (VLP) was introduced. Machine learning techniques were also used to mitigate the LED’s non-linearity issue for an OFDM-based VLC system [24]. A long short-term memory (LSTM) network was also studied for processing random sequential data [25,26,27].

In this work, through a series of experimental investigations, the effectiveness of machine learning techniques in VCSEL-based VLC is investigated. In particular, using a commercial VCSEL and bidirectional long short-term memory (Bi-LSTM)-based machine learning schemes, a high-speed VLC link with a data rate of 13.5 Gbps and a 2 m link distance is demonstrated. The rest of this work is organized as follows. Section 2 presents the characteristics of VCSELs. In Section 3, the applied Bi-LSTM-augmented deep neural network is introduced. Section 4 shows the proof-of-concept demonstration, showing the results of multi-Gbps VCSEL-based VLC. Then, the discussions and conclusions are presented in Section 5.

## 2. Characteristics of VCSEL

### 2.1. Principle of VCSEL

**Figure 1:**
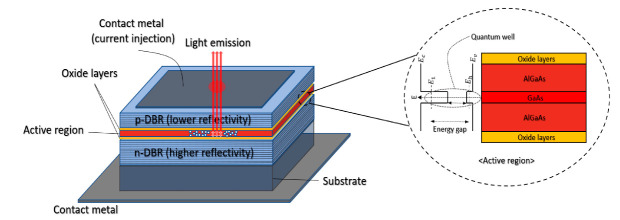
The basic structure of AlGaAs/GaAs VCSEL (**left**). The active region (**right**).

VCSELs emit a laser beam perpendicularly from the top surface. As shown in Figure 1, the structure of the VCSEL can be configured with the top and bottom parts for the current injection, the p-type and n-type mirrors and the active area between them. The distributed Bragg reflectors (DBR) with thin dielectric materials and different reflectivity are stacked. Typically, these materials include layers of semiconductors with alloys or compositions such as AlGaAs, AlGaInAs, InP or InGaAsP [20].

By employing these multilayer reflectors, VCSELs can be used with thin-film lenses, taking advantage of such a thin-layer structure [28]. When the reflectivity of the two mirrors is adjusted differently, the light propagation direction can also be adjusted to diverge toward the less reflective part. The active area makes a quantum well (QW), and, as shown in the inset of Figure 1, by bonding different materials and the proportion of constituent substances, the difference in the energy level between the elements is constructed. Hence, the difference in the energy level generates photons with the corresponding wavelengths. Then, it goes through the oxide layers.

For the infrared VCSELs, DBR pairs of InP/AlGaInAs (850 to 980 nm) or AlGaAs/AlGaAs (1300 nm to 1550 nm) are used [20]. In [29], it was shown that the wave between 650 and 700 nm wavelengths can be obtained from the VCSEL with AlGaInP/GaInP MQW. Additionally, the authors in [30] showed blue VCSELs with five multiple quantum well (MQW) layers emitting the 0.7 mW maximum outputs power at room temperature with a 1.5 mA threshold current and a 3.3 V threshold voltage. Modifying the composition or utilizing different materials for the purpose of enhancing the performance of VCSELs has been actively investigated [31,32]. Further, there have been many studies exploiting the advantages of VCSELs, such as its low cost, low threshold and the narrow beam, which are under investigation in a wide range of applications [33,34,35,36].

### 2.2. Experimental Analysis of VCSELs’ Characteristics

This sub-section presents the characteristics of the off-the-shelf VCSEL (Thorlabs, L670VH1) used for the characterization and VLC performance test. The VCSEL has a typical center wavelength of 670 nm. The threshold current, operating current and corresponding optical output are 0.6 mA, 2.5 mA and 1 mW, respectively. The beam divergence angle (full-width-half-maximum) is 10 degrees.

**Figure 2:**
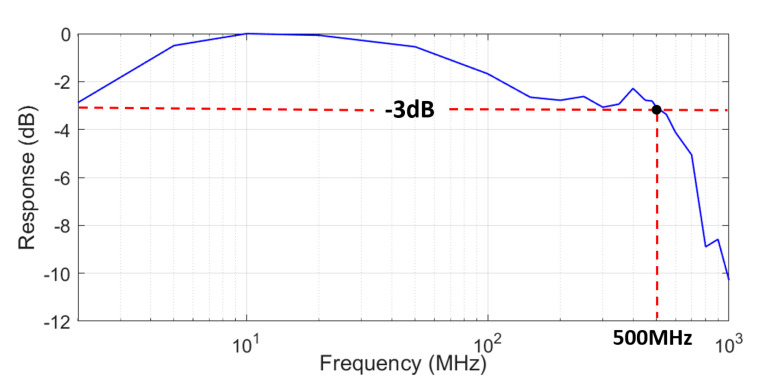
VCSEL (L670VH1) frequency response.

Figure 2 shows the measured frequency response. A high-speed PIN photo-receiver (New-Focus, 1591), arbitrary waveform generator (Tektronics, 70002B), and oscilloscope (Tektronics, DPO70404C) are used for the experiment. The overall measurable bandwidth of the testbed is ~4 GHz. A total of 21 frequency points are measured, which are extracted in the frequency range of 2 MHz to 1 GHz for the frequency measurement. It is confirmed from the measurement that the −3 dB bandwidth of the VCSEL device is 500 MHz. The value is certainly more than an order of magnitude higher than that of typical LEDs, showing the potential of VCSELs for high-speed modulation.

**Figure 3:**
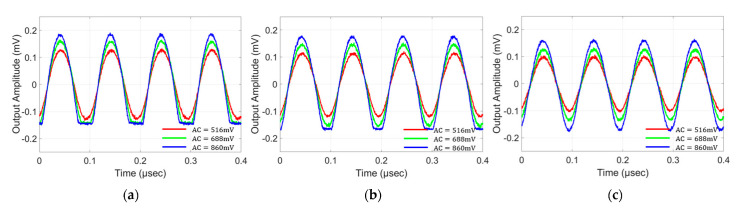
Dynamic response and linearity of the VCSEL; sine wave outputs for (**a**) 1.3 V DC bias, (**b**) 1.4 V DC bias and (**c**) 1.5 V DC bias.

The dynamic response to check the linearity of the VCSEL device and to find the suitable driving condition is shown in Figure 3. The testbed is the same as the frequency response measurement, except that three different levels of sinusoidal amplitudes (516 mV, 688 mV, 860 mV) are applied, while the DC bias is varied from 1.3 V to 1.5 V. The applied frequency is 10 MHz, at which value the dynamic characteristics of the VCSEL device are well observed. The output amplitude from the high-speed photo-receiver is captured by the oscilloscope, and the received signal shape is observed. The other parameters (such as the received optical power) are adjusted to monitor the response from the VCSEL device purely.

Overall, for the higher DC bias, the peak-to-peak levels of the observed output signal are slightly reduced. This is due to the VCSEL’s nonlinear DC characteristics. This tendency can be better seen with the smallest AC signal (516 mV). On the other hand, in the case of a DC bias of 1.3 V (Figure 3a) and an AC signal of more than 688 mV, a clear lower clipping is observed. This is due to the VCSEL’s threshold voltage level being at ~1 V. By increasing the DC bias level to 1.4 V (Figure 3b), it can be seen that the AC signal of 688 mV no longer causes the lower clipping, since the lowest level applied to the VCSEL is higher than the threshold voltage. For the same reason, Figure 3c shows no clipping even with the 860 mV applied to the VCSEL. A higher received signal strength could result in better communication performance; however, it could induce more nonlinear distortion. Additionally, the inclusion of a certain amount of nonlinear distortion while utilizing the high signal level could be beneficial, since it could lead to a higher signal-to-noise ratio (SNR) if the level of nonlinearity is well-compensated, given the other deterioration parameters in the system. The optimum amount of nonlinearity inclusion could depend on the applied communication schemes as well. These postulations are tested in the LSTM-augmented deep neural network in the following sections.

## 3. Long Short-Term Memory Network

**Figure 4:**
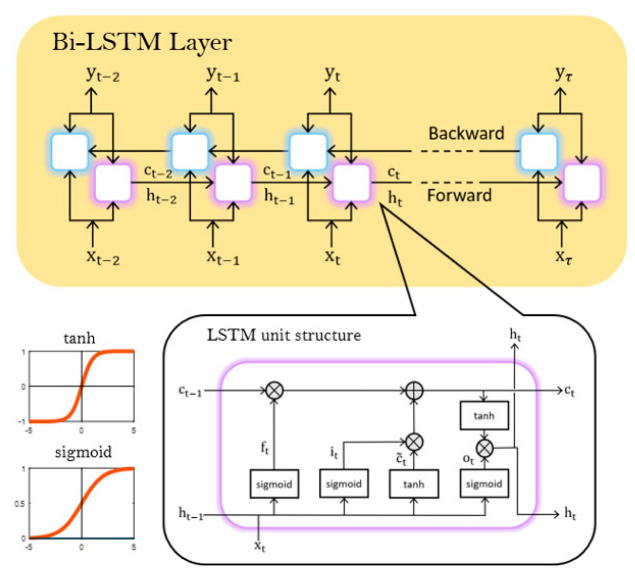
Structure of a Bi-LSTM and LSTM unit and a schematic of a neural network.

An LSTM network, a type of recurrent neural network (RNN), is a deep learning model used for sequential data analysis [26]. LSTMs are designed to overcome the vanishing gradient problem of RNN. For RNN, the longer the data sequence, the smaller the gradient that involves the information used to update the weights and biases, which means that learning is challenging. On the other hand, in the case of LSTMs, there is a cell state vector that may prevent the gradient from vanishing. Using two unidirectional LSTM layers simultaneously to generate a Bi-LSTM network, receiving inputs forwards and backwards effectively increases the amount of information available on the network, possibly leading to better performance [27]. Figure 4 shows the structure of the Bi-LSTM and LSTM units.

Inside the LSTM unit, there are three gates that control the flow of data to better preserve past information: forget gate $f_{t}$, input gate $i_{t}$ and output gate $o_{t}$. Each gate has its own weight and bias. The symbol W and the symbol b denote weight and bias, respectively, and there are two activation functions: the sigmoid function σ and the hyperbolic tangent function tanh. Because past values are constantly used recursively, information needs to be normalized between −1 and 1 using a function.

(1) $$W=\left[\begin{array}{c} W_{f}\\ W_{i}\\ W_{c}\\ W_{o} \end{array}\right],\;b=\left[\begin{array}{c} b_{f}\\ b_{i}\\ b_{c}\\ b_{o} \end{array}\right]$$

(2) $$\sigma \left(\alpha \right)=\frac{1}{1+e^{-\alpha }},\;tanh\left(\alpha \right)=\frac{e^{\alpha }-e^{-\alpha }}{e^{\alpha }+e^{-\alpha }}$$

The forget gate calculates the information to forget in the cell and applies the current input ($x_{t})$ from the current time step and the previous hidden state $h_{t-1}$ from the previous time step to the sigmoid function to calculate the value between 0 and 1.

(3) $$f_{t}=\sigma \left(W_{f}\cdot \left[x_{t},h_{t-1}\right]+b_{f}\right)$$

The input gate determines the data to be updated via the sigmoid function in a similar way to the forget gate. The candidate cell state ${\tilde{c}}_{t}$ is the calculated value between −1 and 1 through the hyperbolic tangent function and is combined with $i_{t}$ to be added to the current cell state $c_{t}$.

(4) $$i_{t}=\sigma \left(W_{i}\cdot \left[x_{t},h_{t-1}\right]+b_{i}\right)$$

(5) $${\tilde{c}}_{t}=tanh\left(W_{c}\cdot \left[x_{t},h_{t-1}\right]+b_{c}\right)$$

The current cell state $c_{t}\;$can be calculated by using the results of the equation above, multiplying $f_{t}$ and the previous cell state $c_{t-1}$ to determine the data to forget and adding the data to update. Then, the part of the cell state to be output is determined by $o_{t}$. Based on the cell state, the parameter to export as the current hidden state $h_{t}$ is specified. This $h_{t}$ is again transferred to the next step and is calculated as the $h_{t-1}$ of the next step.

(6) $$c_{t}=f_{t}\times c_{t-1}+i_{t}\times {\tilde{c}}_{t}$$

(7) $$o_{t}=\;\sigma \left(W_{o}\cdot \left[x_{t},h_{t-1}\right]+b_{o}\right)$$

(8) $$h_{t}=o_{t}\times tanh\left(c_{t}\right)$$

## 4. Proof of Concept Demonstration

### 4.1. Experimental Set-Up

**Figure 5:**
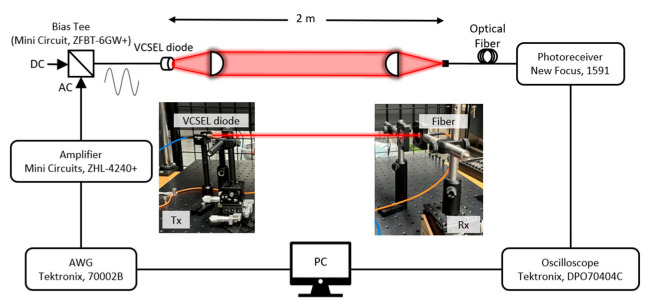
Experimental setup.

The experimental setup for a high-speed line-of-sight VLC link using a VCSEL (Thorlabs, L670VH1) is illustrated in Figure 5. This experiment is performed under ordinary indoor lighting. Two-level pulse amplitude modulation (2-PAM), also known as on-off-keying (OOK), is generated by an arbitrary waveform generator (Tektronix, 70002B) connected to the control PC. Amplified by a wideband amplifier (Mini-Circuits, ZHL-4240+) with a frequency range from 10 to 4200 MHz, the generated communication signal is combined with a suitable DC level by a bias tee (Mini-Circuit, ZFBT-6GW+), according to the measured L-I curve of the VCSEL. Then, the generated electrical signal is applied to the VCSEL, which is transformed into the optical signal. Optomechanical translation stages and appropriate optics for both the transmitter and receiver are used to couple the light into the fiber-pigtailed photoreceiver (New-Focus, model 1591). The distance between the transmitter and receiver is set to 2 m. An oscilloscope (Tektronix, DPO70404C) captures and samples the electrical signal from the photoreceiver and sends the data to the control PC for offline signal processing via MATLAB. Subsequently, the recovered signal and the demodulated bits are processed for the bit-error-rate (BER) performance test.

### 4.2. Communication Test Result

**Figure 6:**
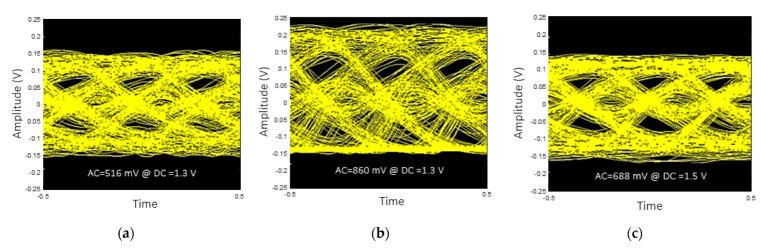
Eye-diagram of the received 2-PAM signal of 3 Gbps applying (**a**) 516 mV AC with 1.3 V DC, (**b**) 860 mV AC with 1.3V DC, (**c**) 688 mV AC with 1.5 DC.

To investigate the VLC performance with various driving conditions in the case of OOK modulation, the BER performance and the received eye-diagrams at a data rate of 3 Gbps, with different peak-to-peak (AC) signals and biases (DC), are investigated. In Figure 6a,b, it can be observed from the eye-diagrams that, for the same DC bias, when the AC value is increased, the lower part is clipped due to it being driven below the threshold voltage of the VCSEL, although the received signal strength (peak-to-peak) increases. The eye-diagram in Figure 6c shows that, by applying a 1.5 V_DC_ bias with a 688 mV peak-to-peak, the received signal strength becomes similar to that in the case of 1.4 V_DC_ with 516 mVpp, but it shows marginally improved eye-opening.

**Figure 7:**
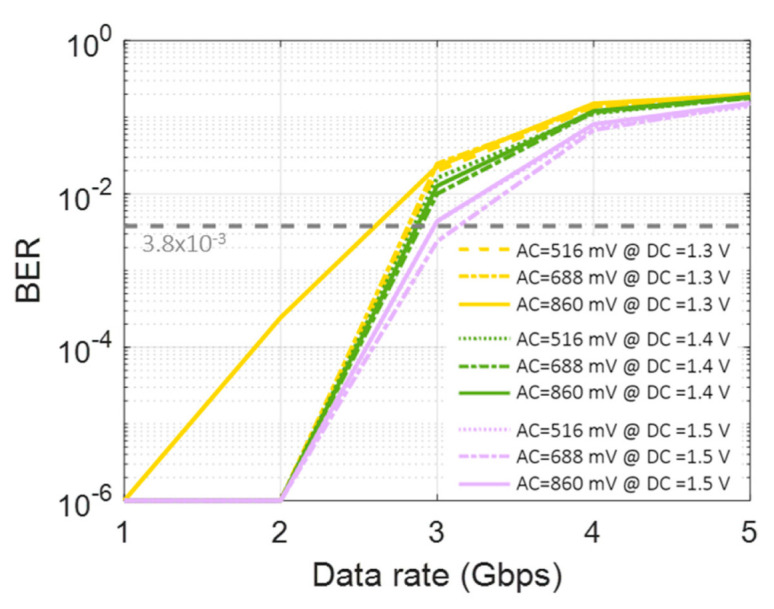
BER performance according to different AC and DC values.

Figure 7 illustrates the BER performances. It is confirmed that the performance tends to improve by increased DC bias, regardless of the AC swing. The worst BER case is when the AC is 860 mV with 1.3 V_DC_, as shown in Figure 6b. The best case is when the AC is 860 mV with 1.3 V_DC_ (as shown in Figure 6b) and the corresponding data rate is ~3 Gbps, satisfying the forward error correction (FEC) with a BER threshold of 3.8 × 10^−3^.

**Figure 8:**
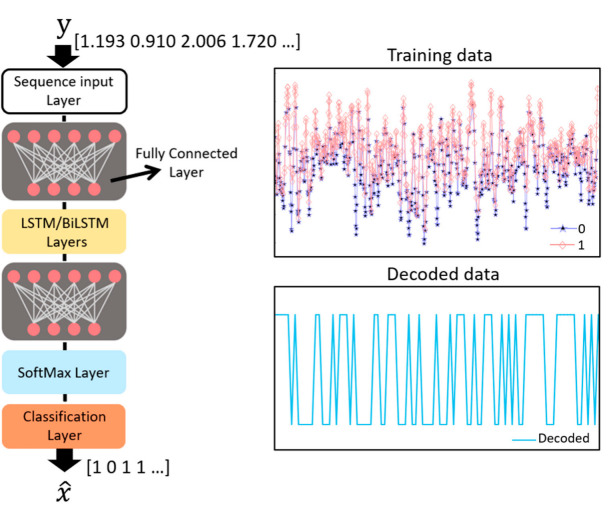
Diagram of the proposed neural network.

Machine learning techniques can be applied to achieve a higher data rate, and the investigated structure is shown in Figure 8. The model consists of a Sequence Input Layer, two Fully Connected Layers, multiple Unidirectional LSTM or Bi-LSTM Layers, and a Classification Layer. The number of LSTM or Bi-LSTM Layers can be optimized empirically, considering the LSTM structure depicted in Figure 4. The model is trained with data generated by the arbitrary waveform generator and the data received from the photoreceiver. The data set is divided into a train set and a test set to train and evaluate the network. The ‘Training data’ shown in Figure 8 indicates the generated data labeled 0 or 1. The symbol y denotes the received aliased data to be recovered, and the symbol $\hat{x}$ denotes the recovered data. The training model is formed through the operation of several layers in the network and a series of computations. Then, the data corrupted by noise and interference are compensated by the network, and it is shown in Figure 8 as ‘Decoded data’. Finally, the decoded data are evaluated by the test set. The training options and the values used for the hyperparameters implemented to train the model are summarized in Table 1.

**Figure 9:**
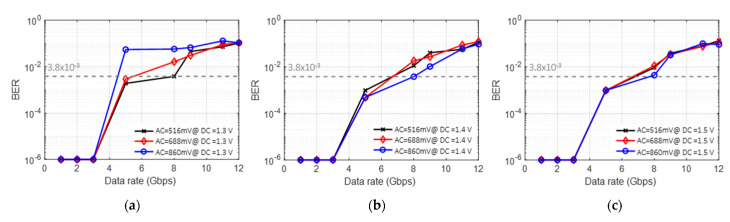
BER performance according to different AC values with a single LSTM network at DC bias of (**a**) 1.3 V, (**b**) 1.4 V, and (**c**) 1.5 V.

To explore the relationship between the waveform shape and the LSTM network, we try to train the machine learning model, which includes a single LSTM layer with 300 LSTM units, and classify the received signal by changing the AC and DC values. In Figure 9, compared to the OOK case in Figure 7, the LSTM shows a noticeable BER performance improvement in all cases. It is especially notable that, for the case of an AC of 860 mV and a DC of 1.4 V (Figure 9b), the achieved data rate is improved to 8 Gbps from the previous OOK result of 3 Gbps.

**Figure 10:**
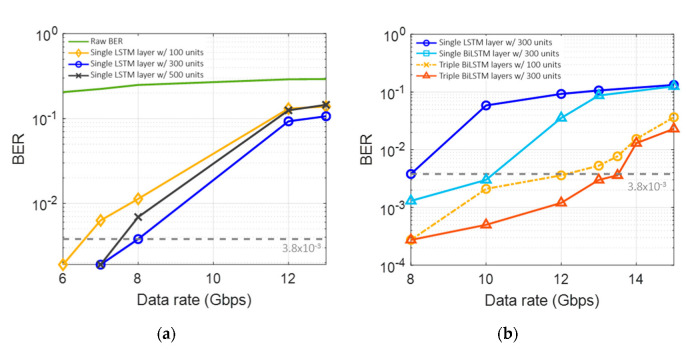
BER performances (**a**) by the number of units, (**b**) by the number of layers and types.

It is also found that there are optimum machine learning configurations for the tested VCSEL-based VLC link. In particular, the type and depth of the layer and the number of units affect the machine learning performance. Such configurations are optimized to further increase the data rate. An AC of 860 mV with a DC of 1.4 V is applied to the VCSEL. Figure 10a shows the BER performance according to the number of units in a single LSTM layer. ‘Raw BER’ means the case without any machine learning method. There is a BER improvement from 100 to 300 units, but the performance is slightly decreased compared to the improvement from 300 to 500 units. The Bi-LSTM layers are also tested, as shown in Figure 10b. It can be seen that the single Bi-LSTM layer outperforms the single LSTM layer, and the triple Bi-LSTM layers show excellent performance, with a data rate of 13.5 Gbps, satisfying the forward error correction (FEC) with a BER threshold of 3.8 × 10^−3^.

## 5. Discussion and Conclusions

Since VCSELs have been developed quickly in recent decades, a number of studies have shown VCSELs’ feasibility in a wide range of applications. In particular, in optical wireless communication and visible light communication, VCSELs are considered one of the most promising light sources due to their low manufacturing cost, high modulation bandwidth and straightforward operation and management. In this work, to meet the multi-Gb/s data rate requirements imposed on the next-generation wireless communication system, the sub-GHz modulation bandwidth of the off-the-shelf VCSELs was successfully compensated for by utilizing the bidirectional long short-term memory-based machine learning scheme. To the best of the authors’ knowledge, the highest single channel data rate (13.5 Gbps) with a 2 m link distance from a cost-effective commercial VCSEL device was demonstrated. Additionally, the spectral efficiency (data rate/bandwidth) is much higher than that in previous work. The best result reported in a previous work [21] using an OFDM scheme showed 2.4 bps/Hz; however, the method introduced in this work has an efficiency of 27 bps/Hz, which is ~11 times higher than that previously reported. Future work includes further improving the data rate by applying other modulation and multiplexing schemes and exploring other effective machine learning techniques for VCSEL-based visible light communication networks, considering the efficiency of the machine learning process at the same time. Additionally, in-depth analytic investigations of the chaos-theory-based algorithms with less complexity in order to overcome the complex structure of modern deep neural networks are potential research topics.

## Figures and Tables

**Table 1:** The training options and the hyperparameters’ values.

Parameter	Values
Optimizer	Adam
Learning Rate	0.001
Number of Epochs	200
Gradient Threshold	1
Shuffle	Once
Execution Environment	GPU
Sequence Length	Longest
Loss Function	Cross-entropy

## Data Availability

The data presented in this study are available on request from the corresponding author and are on his personal website.

## Abbreviation

AC	alternating current
AI	artificial intelligent
AML	adversarial machine learning
BER	bit-error-rate
Bi-LSTM	bidirectional long short-term memory
DBR	distributed Bragg reflector
DC	direct current
DCO-OFDM	direct current-biased orthogonal frequency division multiplexing
FEC	forward error correction
LD	laser diode
LED	light-emitting diode
L-I	light output-current (I)
L-PAM	L-level pulse amplitude modulation
LSTM	long short-term memory
ML	machine learning
MQW	multiple quantum well
OOK	on-off-keying
OWC	optical wireless communication
QAM	quadrature amplitude modulation
QW	quantum well
RF	radio frequency
RNN	recurrent neural network
SNR	signal-to-noise ratio
VCSEL	vertical-cavity surface-emitting laser
VLC	visible light communication
VLP	visible light positioning system
